# Need for close interdisciplinary communication after endoscopic endonasal surgery to further personalize postoperative radiotherapy in sinonasal malignancies

**DOI:** 10.3389/fonc.2023.1130040

**Published:** 2023-02-28

**Authors:** Florent Carsuzaa, Valentin Favier, Marco Ferrari, Mario Turri-Zanoni, Rossana Ingargiola, Anna Maria Camarda, Lise Seguin, Giacomo Contro, Ester Orlandi, Juliette Thariat

**Affiliations:** ^1^ Department of Otorhinolaryngology – Head and Neck Surgery, University Hospital of Poitiers, Poitiers, France; ^2^ Department of Otorhinolaryngology – Head and Neck Surgery, University Hospital of Montpellier, Montpellier, France; ^3^ Section of Otorhinolaryngology – Head and Neck Surgery, University of Padova, “Azienda Ospedale Università di Padova”, Padova, Italy; ^4^ Division of Otorhinolaryngology – Head and neck surgery, Department of Biotechnology and Life Sciences, University of Insubria, ASST Sette Laghi University Hospital, Varese, Italy; ^5^ Radiation Oncology, Clinical Department, National Center for Oncological Hadrontherapy (CNAO), Pavia, Italy; ^6^ Radiation Oncology, Centre François Baclesse: ARCHADE, Caen, France

**Keywords:** sinonasal malignancies, radiotherapy, surgery, skull base tumors, interdisciplinary

## Introduction

Sinonasal malignancies represent 3% of the head and neck cancers and have an incidence of 1/100,000 persons a year. Numerous epithelial and non-epithelial histologic subtypes are described in this localization: squamous cell carcinomas in 50% of sinonasal tumors, intestinal-type adenocarcinomas (ITAC) in 13%, mucosal melanomas in 7%, olfactory neuroblastomas in 7%, adenoid cystic carcinomas in 7%, undifferentiated carcinomas in 3%, and other very rare histologies in 13% (1–3). Most sinonasal malignancies are locally aggressive diseases. Five-year survival rates vary between 30–60%, with local recurrence as the main cause of death. Oncologic outcomes indeed vary widely with histological subtype: 5-year overall survival rates are 50-66.2% for squamous cell carcinomas, 60.0-72.7% for ITAC, 30.0%-35.7% for melanomas, 70.0-94.0% for olfactory neuroblastomas, 35.0-82.2% for undifferentiated carcinomas, and 87.7% for adenoid cystic carcinoma (4).

The management of sinonasal malignancies has undergone several changes in recent years. Surgery was initially performed through open approaches with complication rates of 33–42% and 4% of mortality. It has evolved toward minimally-invasive endoscopic surgery. Such strategy has allowed non-inferior oncological local control (LC) and lower morbidity (about 7% of complications, overall) (5, 6). Endoscopic surgery is now the mainstay of treatment in most sinonasal tumors. Radiotherapy (RT) is often necessary as an adjuvant modality, with or without chemotherapy, following surgery to optimize local control. Such key decisions for the patients should be made at multidisciplinary staff meetings before each important step (surgery, radiotherapy, etc).

When a tumor involves the skull base and paranasal sinuses, surgeons and radiation oncologists faced with several anatomical challenges: 1) most of the anatomical landmarks are difficult to identify on an CT imaging basis due to a soft-tissue CT density (but also on MRI) and a combination of invasion and compression/displacement of normal anatomical structures; 2) sinonasal tumors are often pedicled, with a larger “polyp-like” intraluminal compartment (i.e.: the portion of tumor which is growing in the sinonasal air spaces) which is not invasive for surrounding structures; 3) postoperatively, most of the anatomical landmarks are removed (e.g.: turbinates, sinuses walls, crista galli…); 4) during RT administration, some changes in the mucosal thickness and modification of collected fluid can contribute to the amount of aeration inside the paranasal sinuses with a significant impact on dose distribution on target volumes and organs at risk (7). Of course, structures adjacent to sinonasal regions are also at risk and need to be preserved. However, it is necessary to guarantee the effectiveness of the treatment allowing good LC. Orbital preservation best represents the compromise made to achieve acceptable oncological results while maximizing functional outcomes (8). Therefore, sinonasal tumors are indeed an example of multidisciplinarity and should be best performed at high volume centers (9), a statement that of course is challenged by the rarity of these tumors and their variable presentations and histologies.

## Interdisciplinary communication

Endoscopic surgery is a major change for radiation oncologists. To that extent, it should be well documented and understood to ensure tumor control. Mutual understanding of endoscopic surgery might also be a great opportunity to not only reduce surgical morbidity but also radiation-induced morbidity.

Planning post-operative radiotherapy (PORT) in sinonasal malignancies is a complex task. It is inherently interdisciplinary, requiring mutual understanding of the various reports made during the course of treatment: the imaging report should be well understood by the surgeon; in particular, MRI has been reported to overestimate tumor extensions, suggesting that the surgeons should be able to discuss with radiologists before surgery (10). It also allows feedback from surgeons to radiologists based on perioperative observations as a way to make imaging reports more usable by the practicing surgeons, recognizing the implications of imaging reports. Perioperative observations could be enriched by neuronavigation images to correlate tumor areas with radiologist’s assessment and also with radiation oncologists to transpose operate findings onto postoperative the PORT planning CT scanner.

Endoscopic endonasal resection led to tumor disassembling into smaller tissue fragments, which may measure a few centimeters only. Each of these fragments is typically annotated and oriented by the surgeon (11). Standardization of oriented surgical sampling and the way the anatomical areas are resected is key. Operative reports may either refer to a graphical or textual list of the resected anatomical structures. They are the basis for a common language for all specialties involved in the management of patients with sinonasal tumors. The pathologist can locate safe tissue fragments, tumor sub-volumes, and can report on the quality of tumor margins and additional surgical margins. Their analysis is essential to qualify the quality of margins and further the dose defined by the radiation oncologists.

Finally, historadiological correlations are needed to further locate each fragment onto the successive planar images of the planning radiotherapy scanner. The volumes to be irradiated are usually determined from the imaging performed pre-operatively, the operative and pathological reports and knowledge of anatomical extensions. At the era of endoscopic surgery, communication between the surgeon and radiation oncologist is critical to accurate RT volume and dose definitions so as to ensure local tumor control (12).

The whole process requires continuous interdisciplinary communication. This is a strong prerequisite to further optimize RT in a way that could reduce irradiation volumes, as local failure cannot be an option when customizing treatments toward less morbidity from RT.

## Toward a better use of scheme and navigation

A schematic anatomic 3D drawing was designed by Bastier and de Gabory and validated as a tool to help clinicians to report the surgical and pathological results of sinonasal malignancy removal (11). Those not familiar with this figure may also use the corresponding list table of anatomical fragments that is necessarily report on the pathology report following surgical minimally-invasive and annotated resection. It indicates the extension of the surgical resection and locates all the histological specimens. It helps in understanding the position and relationship between each sample, which is very helpful since many fragments are removed from the same anatomical structure during the surgery for sinonasal malignancies. It demonstrates tumor invasion within the resected structures and shows the free margins. The use of this scheme makes it possible to improve the communication between the various stakeholders and in particular the pathologist’s understanding of the position of the various tumor portions and the radiation oncologist’s understanding of the areas most at risk of relapse. However, this scheme is a fragmented view of the sinonasal anatomical spaces, which can make it difficult to represent tumors especially in the borderline areas.

Advances in in-room imaging using surgical navigation systems can help surgeons intraoperatively and might further improve the rate of safe margins and may further improve oncologic outcomes (13). The accuracy of new generation navigation systems is now < 1 mm (14). Given the complex anatomical structures that lie in close proximity to critical structures such as optic nerves, orbits and their content, optic chiasm, pituitary gland, internal carotid artery, cranial nerves and brain, this tool helps to reduce morbidity of the surgery. The use of surgical navigation also allows the surgeon to combine the macroscopic view with a precise CT/MRI location as well as to better understand the tumor implantation area. In the future, data acquired during intraoperative navigation could be coupled with the pre- and postoperative imaging to enhance accuracy. The surgeon could then contour the area of the tumor implantation during the surgery with a navigated pointer and this contouring could automatically be transferred to the postoperative imaging in order to facilitate understanding of the volumes at risk by the radiation oncologist.

## Radiotherapy planning

The low morbidity of endoscopic sinonasal surgeries can be combined with modern irradiation modalities to promote minimally invasive and maximally effective therapeutic options. PORT planning of sinonasal malignancies, in its delineation step, requires virtual geolocation transfer of sinonasal tumor from preoperative views to postoperative axial CT in RT practice. This is of pivotal importance not only for optimal delineation of the initial macroscopical disease, but also for the selection, along with histopathological data, target volumes at risk of harboring microscopic disease.

Given that toxicities are cumulative between surgery and RT, these findings point the opportunity to improve patient care and avoid therapeutic escalation. Two trials are currently investigating de-escalation in high-dose volumes. The French “SinocaRT” randomized phase II trial compares dose-painting intensity-modulated radiation therapy (IMRT) versus standard IMRT. The GORTEC 2016-02 phase III SANTAL trial NCT02998385, investigates the use of cisplatin in addition to PORT; it includes an arm with proton therapy. Accrual will end in 2023 with more than 260 patients. An American trial (NCT01586767) currently investigates the efficacy of proton therapy on local control rate and the toxicity compared to IMRT in the treatment of locally advanced sinonasal malignancy. Such precision RT includes other constraints. Using minimally-invasive endoscopic technique, the resected tumor and surrounding anatomical structures are small and characterized by a complex shape. Therefore the dose distribution may be geometrically complex with areas of steep dose gradients (in the order of 1-2 Gy/mm). Complex dose geometry requires quality assurance processes that consists in assessing whether an optimally planned radiotherapy can indeed be delivered accurately as planned. Moreover, the resected sinonasal areas have complex air-soft tissues/mucosae-bone interfaces (regardless of the type of surgery). These interfaces may change from day to day because sinonasal cavities are variably filled in with secretions. Secretions can change in quantity and density over the treatment course along the beam paths and might result in inaccurate dose delivery.

Improvements to facilitate dose-painting RT might come from optimized reporting during the endoscopic surgery procedure. The surgical navigation system using a digital pointer on CT images may be exploited further by radiation oncologists when planning PORT. If images and pointing information can be stored, these images may be co-registered and transferred on postoperative radiotherapy planning CT. 3D tumor reconstruction and printing might also be another intriguing option. At present, it has been used only for educational purposes with students or as some information aid for patients (15, 16). It could provide a postoperative basis to improve understanding of positive margins on the tumor anatomy preoperatively. However, it is time consuming for the surgeons and pathologists, and this may be selected for complex cases until automated solutions will be available.

The sinonasal structures are anatomically complex and small and therefore difficult to precisely delineate on CT by the radiation oncologist. Due to the lack of specific sinonasal atlases identifying endoscopic resection fragments, the anatomic uncertainties and the level of confidence of using histosurgical mapping by radiation oncologists may limit the use of dose-painting IMRT in daily practice (17). Even more, this could be limiting for proton therapy due to the sharp dose profiles usually achieved and a higher sensitivity to uncertainties compared to IMRT, requiring caution to avoid unintended hot spots. It is therefore necessary to create tools allowing radiation oncologists to better navigate in postoperative imaging. Researches in this area should focus on automatization of tumor and sinonasal anatomy representation (e.g.: automatic recognition of anatomical spaces and segmentation on CT/MRI images).

## Conclusion

As surgery-related morbidity has been reduced over the years by the contribution of endoscopic-assisted endonasal surgery, an attempt should be made to limit postoperative sequelae of RT and improve oncological outcomes by more precise targeting of areas at risk of tumor extension. RT is associated with severe middle- and long-term toxicities, suggesting efforts should be made by the scientific community to reduce its morbidity. The first major challenge, although there are no clinical studies on this point, is to be able to reduce the irradiated volumes to reduce radiation-induced morbidity and without taking an additional risk of local relapse. Closer communication between surgeons, pathologists and radiation oncologists, with the help of specifically designed tools, is mandatory to achieve the next step in sinonasal malignancy management. This may allow a better personalization of postoperative treatment to improve LC and reduce the morbidity of combined surgical and RT.

## Author contributions

Conceptualization: FC, VF, JT. Original writing: FC, VF, JT. Reviewing original manuscript: all authors. All authors contributed to the article and approved the submitted version.
